# Thicker three-dimensional tissue from a “symbiotic recycling system” combining mammalian cells and algae

**DOI:** 10.1038/srep41594

**Published:** 2017-01-31

**Authors:** Yuji Haraguchi, Yuki Kagawa, Katsuhisa Sakaguchi, Katsuhisa Matsuura, Tatsuya Shimizu, Teruo Okano

**Affiliations:** 1 Institute of Advanced Biomedical Engineering and Science, TWIns, Tokyo Women’s Medical University, 8-1 Kawada-cho, Shinjuku-ku, Tokyo 162-8666, Japan; 2 Institute for Nanoscience and Nanotechnology, Waseda University, 2-2 Wakamatsu-cho, Shinjuku-ku, Tokyo 162-8480, Japan; 3 School of Creative Science and Engineering, TWIns, Waseda University, 2-2 Wakamatsu-cho, Shinjuku-ku, Tokyo 162-8480, Japan

## Abstract

In this paper, we report an *in vitro* co-culture system that combines mammalian cells and algae, *Chlorococcum littorale*, to create a three-dimensional (3-D) tissue. While the C2C12 mouse myoblasts and rat cardiac cells consumed oxygen actively, intense oxygen production was accounted for by the algae even in the co-culture system. Although cell metabolism within thicker cardiac cell-layered tissues showed anaerobic respiration, the introduction of innovative co-cultivation partially changed the metabolism to aerobic respiration. Moreover, the amount of glucose consumption and lactate production in the cardiac tissues and the amount of ammonia in the culture media decreased significantly when co-cultivated with algae. In the cardiac tissues devoid of algae, delamination was observed histologically, and the release of creatine kinase (CK) from the tissues showed severe cardiac cell damage. On the other hand, the layered cell tissues with algae were observed to be in a good histological condition, with less than one-fifth decline in CK release. The co-cultivation with algae improved the culture condition of the thicker tissues, resulting in the formation of 160 μm-thick cardiac tissues. Thus, the present study proposes the possibility of creating an *in vitro* “symbiotic recycling system” composed of mammalian cells and algae.

Green plants and algae synthesize adenosine triphosphate (ATP) and reducing nicotinamide adenine dinucleotide phosphate (NADPH), and excrete O_2_ by using light energy and H_2_O^1^. In the carbon-fixation cycle (Calvin cycle), glyceraldehyde-3-phosphate (G3P) is synthesized from CO_2_ and H_2_O by utilizing ATP and NADPH. Nutrients including carbohydrates are synthesized from G3P. On the other hand, animals including humans consume the O_2_ and nutrients in respiration and synthesize ATP. Carbon dioxide, produced by respiration, is reused as a core component in photosynthesis. In agriculture, animal excreta, manure, has been used as a valuable source of nitrogen for crop plants. Glutamate is synthesized from 2-oxoglutarate and ammonia, which is a metabolite of animals, by green plants and algae. Glutamine is synthesized from glutamate and ammonia. Other amino acids are synthesized by transamination. Animals use these amino acids. On the earth, all organisms are in a “symbiotic relation”, and the natural environment has built a “recycling society” by utilizing their carbon-nitrogen-oxygen cycles.

Primary cells isolated from biological tissues/organs can proliferate *in vitro* and are commonly utilized as cell sources in regenerative therapy. The number of cell-based regenerative and tissue-engineered therapies that can be clinically applied to repair damaged tissues/organs has rapidly expanded in the past few years^2^,^3^. Our laboratory has developed a temperature-responsive culture surface, and reported on the tissue engineering methodology, “cell sheet technology”, that makes use of this culture surface^4^. Because harvested cell sheets maintain their cell-cell junctions, cell surface proteins, and the extracellular matrix (ECM), cell-dense three-dimensional (3-D) tissues can be created by simply layering those cell sheets without any 3-D scaffolds to create tissues that engraft more efficiently onto target tissues without the need for suture^5^,^6^,^7^,^8^,^9^. The transplantation of cell sheets into various animal models with damaged tissues enabled the recovery of their original tissue functions, and numerous clinical studies using single- or multi-layered cell sheets have already been performed successfully^7^,^8^,^9^,^10^,^11^,^12^,^13^,^14^,^15^,^16^,^17^,^18^.

Recently, 3-D culture systems have become a focus in the field of cell biology^19^. The cellular environments of two-dimensionally (2-D) cultured cells are significantly different from the 3-D cultured cells, and these differences affect the gene expression and biochemical activity of those cells. Importantly, a 3-D culture system much more closely resembles *in vivo* situations^20^,^21^. A functional 3-D tissue, which surrogates actual living tissues, is also valuable as an *in vitro* tissue model to assess the efficacy and cytotoxicity of candidate drugs. An optimal 3-D tissue model can be expected in the fields of pharmacology and toxicology.

While cell-dense 3-D tissues can be easily created by the simple layering of cell sheets^5^,^6^, the ischemic environment makes the creation of thicker tissues difficult. The thickness limitation of 3-D tissues without vascular networks is approximately 40–80 μm^22^,^23^. Severe hypoxia/undernutrition within thicker multi-layered cell sheet-tissues without vascular networks is likely, which can induce tissue damage^22^,^23^,^24^. Thus, the thickness limitation of a viable tissue depends on O_2_/nutrient gradients. Moreover, noxious metabolites including lactate and ammonia have been reported to be toxic to cultured cells^25^. The accumulation can also induce cell death within thicker tissues. Cell death within thicker tissues can be controlled by supplying sufficient O_2_/nutrients and removing the metabolites. The creation of thicker tissues like organs is a long-standing goal in the field of tissue engineering, and the transplantation of thick tissues offers hope for more efficient therapies and the enlargement of the range of applications for regenerative therapies. Additionally, the thicker native-like tissue will be an optimal *in vitro* tissue model.

Here we report about the co-cultivation of mammalian cells and algae to simplify the creation of thicker tissue. This report shows that the co-cultivation method has some potential in the fields of cell biology, tissue engineering, and regenerative medicine.

## Results

### Detection of O_2_ production from algae

**Figure 1:**
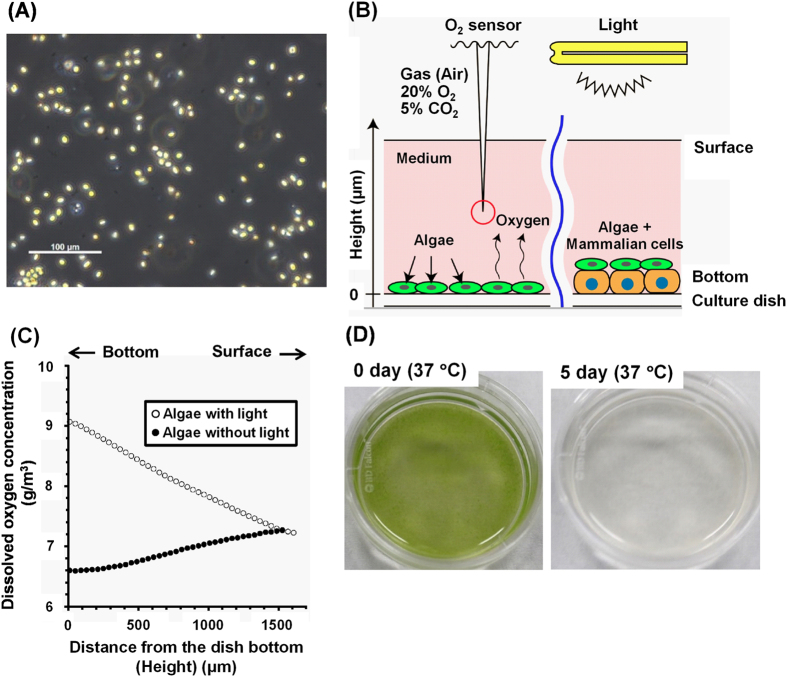
Oxygen measurement system and algae, *Chlorococcum littorale*. A photograph of the algae (**A**). The schematic illustration of the system for oxygen concentration measurement (**B**). Representative oxygen concentration profiles plotted against the height from the bottom of the dish used for culturing the algae in an M199-based culture medium with/without light at 30 °C (**C**). The photographs of the algae after 0-day- (left) and 5-day-cultivation (right) at 37 °C on 35-mm polystyrene culture dishes (**D**).

An investigation was conducted to determine whether algae, *Chlorococcum littorale*^26^ (Fig. 1A), could be cultured and produce O_2_ within a culture media and temperature conditions optimized for mammalian cells. The algae were suspended in a medium for mammalian cells, and oxygen concentration in the medium was measured by an analysis system, which was developed recently^27^,^28^,^29^, shown schematically in Fig. 1B. Oxygen concentration was greater than the saturation concentration on the surface at 30 °C (Fig. 1C) and 37 °C (data not shown). The highest oxygen concentration was detected in the bottom of the culture dish, and the concentration decreased toward the surface, showing that the algae actively produced oxygen. However, after cultivation for 1 day at 37 °C, the algae showed a drastic decrease in oxygen production (data not shown), suggesting damage to the algae at such high temperatures. The algae, which were cultured at 37 °C for 5 days, showed a bleaching phenomenon (Fig. 1D). On the other hand, after cultivation at 30 °C for 1 day, the algae continued to produce oxygen, which was comparable with the precultivation (data not shown). Typically, many algae are cultured at 30 °C or less^30^, and healthy mammalian cells and cell sheets can also be functionally cultured at 30 °C (unpublished observation). Therefore, in the following experiments, the algae and mammalian cells were cultured at 30 °C (Table 1). Under dark conditions, oxygen concentration decreased to less than the concentration at the surface of the culture dish, namely, the saturated oxygen concentration (Fig. 1C), showing that the algae consumed oxygen under these conditions. It was suggested that the algae’s metabolism changed from photosynthesis to respiration by the absence of light. In this system, oxygen supply could be controlled by light.

### Co-cultivation of mammalian cells with algae

**Figure 2:**
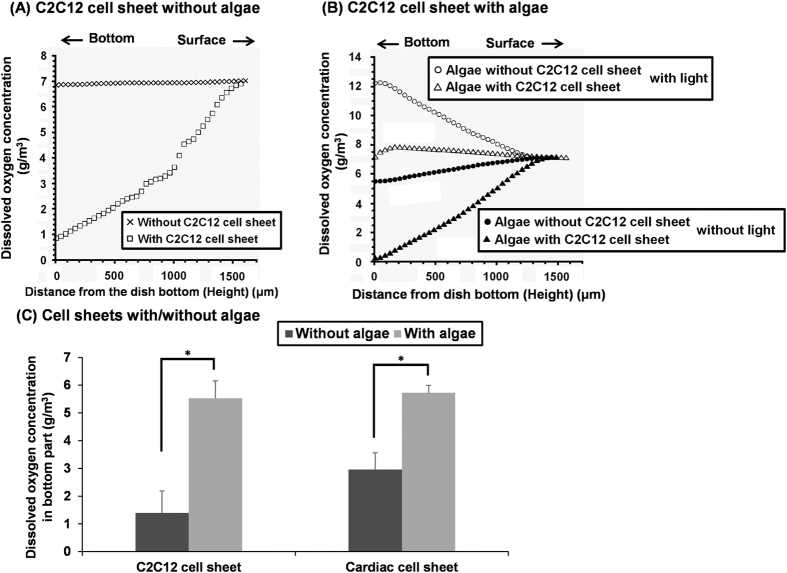
Cultivation of single-layer C2C12 cell sheet or rat cardiac cell sheet with/without algae, *Chlorococcum littorale*. Representative oxygen concentration profiles plotted against the height from the bottom of a culture dish used for culturing a C2C12 cell sheet without (**A**) and with the algae (**B**). In (**B**) panel the experiments were performed with/without light. The dissolved oxygen concentrations at the bottom of the dish, in which a C2C12 cell sheet or a rat cardiac cell sheet was cultured with/without the algae, are the average of the obtained data, which were the average values from the three points measured (n = 2) (**C**). *p < 0.05.

Next, the oxygen consumption of mammalian cells was measured. In a culture dish without a C2C12 cell sheet, there was very little difference between the oxygen concentration at the surface and the bottom of the dish (Fig. 2A). However, in cultivation with a cell sheet the saturated oxygen concentration observed at the surface gradually decreased toward the bottom (Fig. 2A), showing that the confluent C2C12 cells actively consumed oxygen. When only the algae were present on the bottom without a C2C12 cell sheet, as shown in Fig. 1B (left), a high oxygen concentration (more than the saturated oxygen concentration level) was observed under light (Fig. 2B). However, the oxygen concentration under dark conditions was noted to be significantly lower (Fig. 2B). The result was similar to that of Fig. 1C. In the co-cultured area of the dish with a cell sheet and algae under light, shown schematically in Fig. 1B (right), the oxygen concentration in the bottom was similar to that on the surface (Fig. 2B), showing the algae actively produced oxygen, even in co-cultivation. Under dark conditions, the oxygen concentration in the co-culture area decreased significantly to the zero level (Fig. 2B). Under dark conditions, algae also consumed oxygen through respiration. Therefore, in the absence of light co-cultivation oxygen consumption was greater than the oxygen consumption in the cultivation of mammalian cells alone (Fig. 2A,B). In the same co-cultivation system of algae and another mammalian cell type, rat cardiac cells, similar oxygen profiles were also observed (data not shown). Under light, oxygen concentrations at the bottom of a C2C12 cell sheet or a rat cardiac cell sheet with algae were significantly higher than those at the bottom of same cell sheets alone (Fig. 2C). These results showed that the mammalian cells and the algae could be successfully co-cultured, and the algae were able to produce oxygen even under the co-cultivation conditions.

### Co-cultivation of mammalian cells and algae for creating thicker tissues

**Figure 3:**
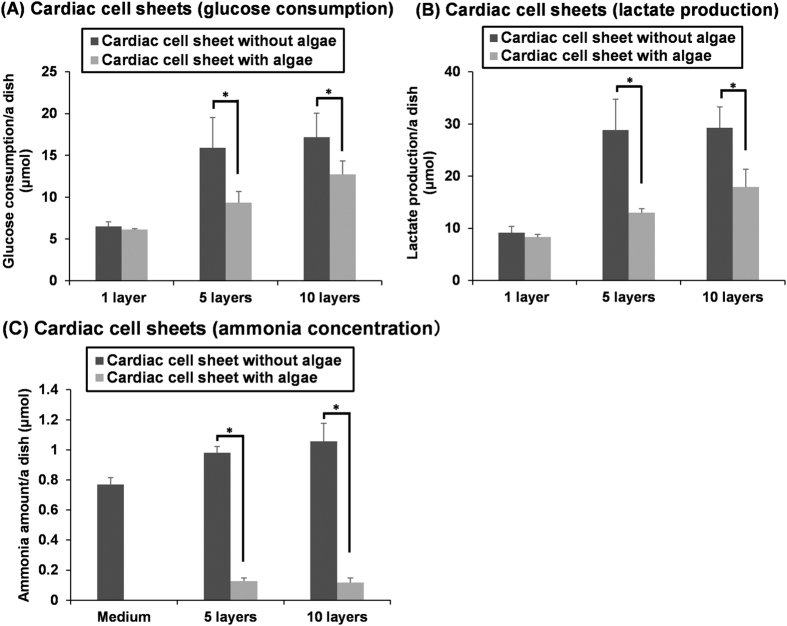
Cell metabolism of single- or multi-layered rat cardiac cell sheets with/without algae, *Chlorococcum littorale*. Comparisons of glucose consumption (**A**) and lactate production (**B**), and ammonia amounts (**C**) in the medium for 24 h-cultivation by single- or multi-layered cardiac cell sheets with/without the algae. Single- and five-layered cell sheets: n = 3; ten-layered cell sheets: n = 5. *p < 0.05.

To create thicker cardiac tissues, five or ten rat cardiac cell sheets were layered with or without algae as shown in Supplementary Fig. 1A, and the multi-cell layered tissues were then co-cultivated in a chamber under light conditions (Supplementary Fig. 1B). Firstly, cell metabolism within the tissues was examined. The glucose consumption and lactate production of a single-layer cardiac cell sheet hardly changed during co-cultivation (Fig. 3A,B). However, multi-layered cardiac cell sheets significantly decreased glucose consumption and lactate production in co-cultivation with algae (Fig. 3A,B). The ratio of lactate production to glucose consumption (L/G ratio) of cells was used as an index of the state of aerobic or anaerobic respiration. When all pyruvates, which are the final product of glycolysis, are converted to lactates in anaerobic respiration, the L/G ratio is two. On the other hand, in aerobic respiration pyruvate enters the mitochondrion to be fully oxidized by the tricarboxylic acid (TCA) cycle, and thus the L/G ratio becomes less than two. In the case of a single-layer cardiac cell sheet, the L/G ratio was 1.40 ± 0.07 (n = 3), showing partial aerobic respiration (Table 2). The L/G ratio [1.36 ± 0.07 (n = 3)] tended to be slightly lower in co-cultivation with algae (Table 2). In the single-layer cell sheet, because the tendency for aerobic respiration was so strong, the significant change of the L/G ratio may not have been detected due to the supply of oxygen from the algae. The L/G ratio of five- or ten-layered cardiac cell sheets was 1.82 ± 0.04 (n = 3) or 1.71 ± 0.14 (n = 5), respectively, showing that anaerobic respiration was higher within the thicker tissues (Table 2). However, the L/G ratios of those same five-layered or ten-layered cell sheets were decreased significantly to 1.41 ± 0.20 (n = 3) or 1.40 ± 0.14 (n = 5), respectively, in co-cultivation with algae, and the values were comparable with that of the single-layer cell sheet (Table 2). The result suggests that oxygenation within the multi-layered cell sheets occurred by the co-cultivation with algae as like the single-layer cell sheet, and a change from anaerobic respiration to partial aerobic respiration with the oxygen supply. Approximately 30 molar ATP are produced from the consumption of one molar glucose without lactate production in aerobic respiration, while only two molar ATP are produced by the consumption of one molar glucose in anaerobic respiration^1^. It was thought that the significant decrease in glucose consumption and lactate production of multi-layered cardiac cell sheet-tissues in co-cultivation with algae was related to effective ATP production in aerobic respiration.

Secondly, ammonia in the culture supernatant of multi-layered cardiac cell sheets was analyzed. Because the culture medium contained fetal bovine serum (FBS) and amino acids, including L-glutamine, ammonia was detected in the culture medium (Fig. 3C). The concentration of ammonia was increased by the cultivation of multi-layered cell sheets (Fig. 3C). However, ammonia within the culture medium of the multi-layered cardiac cell sheets was significantly lower [less than one-seventh (five-layered cell sheets) or one-eighth (ten-layered cell sheets)] in the co-cultivation with algae (Fig. 3C). Ammonia is produced by the degradation of amino acids. While ammonia is not used by animal cells, algae can use ammonia for producing amino acids. It was suggested that the algae used the ammonia within the culture medium to produce amino acids, resulting in the significant decrease in ammonia. Ammonia is an important source of nitrogen for the growth and survival of algae. Although algae can synthesize ammonia from atmospheric nitrogen through nitrogenase, eight molecules of ATP are used for the synthesis of one molecule of ammonia. Thus, the deficiency of ATP or the accumulation of adenosine diphosphate (ADP) inhibits nitrogenase activity. Ammonia is important for algae, so NH_4_Cl (50 μM) was supplied within the culture medium for the algae used in this study.

**Figure 4:**
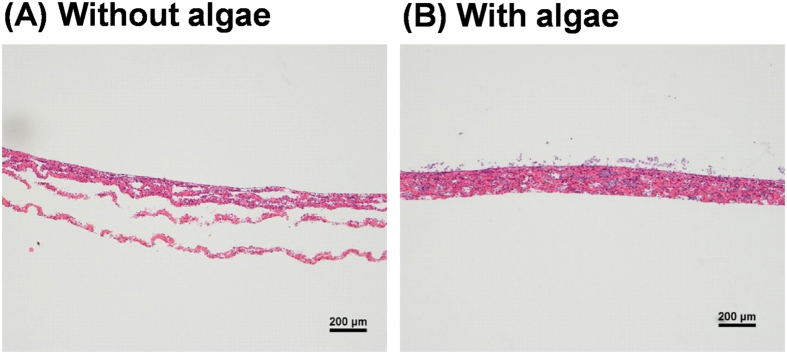
Histological observation of multi-layered rat cardiac cell sheets with/without algae, *Chlorococcum littorale*. The photographs are a histological observation with hematoxylin-eosin staining of five-layered cardiac cell sheets without (**A**) or with (**B**) the algae after a 3-day cultivation, respectively. Three independent experiments were performed and all the experiments showed similar results. The representative photographs are shown in the figure.

Thirdly, the condition of the cells within thicker cardiac tissues and the effect of the co-cultivation with algae were analyzed histologically. Five-layered cardiac cell sheets showed histologically damaged tissues with delamination of the cell sheets on the bottom, which was directly contacted with the dish (Fig. 4A). However, in the five-layered cell sheets with algae, viable stratified tissues containing algae were observed (Fig. 4B). Thicker cardiac tissues approximately 160 μm could be created by the co-culture system (Fig. 4B).

**Figure 5:**
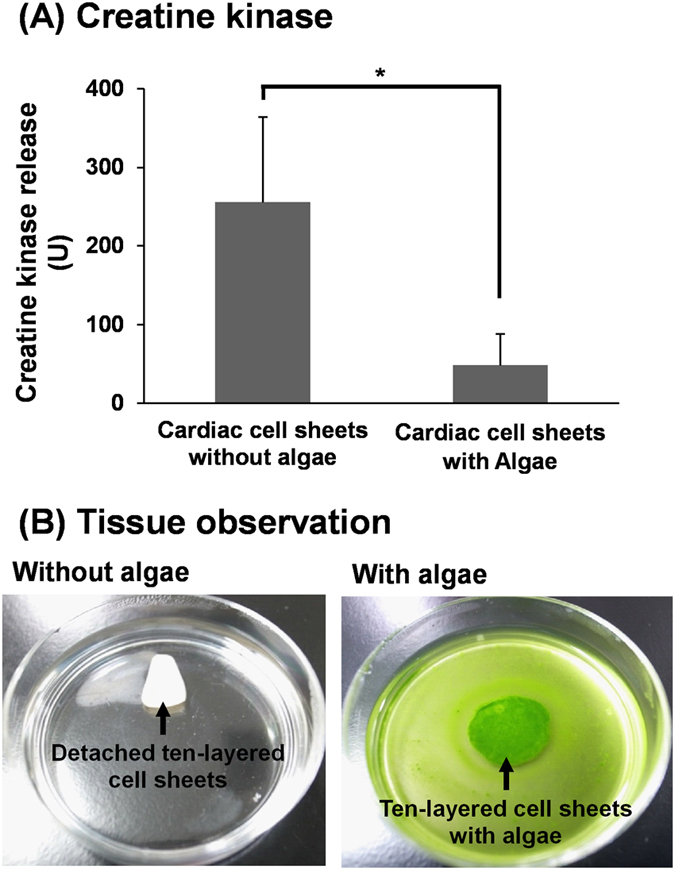
Cytotoxicity assessment of multi-layered rat cardiac cell sheets with/without algae, *Chlorococcum littorale*. The total creatine kinase (CK) release from ten-layered rat cardiac cell sheets with/without the algae during 4 days of cultivation is shown (**A**, n = 5). The release of CK was reduced to less than one-fifth by cocultivation with algae. *p < 0.05. Photographs of ten-layered rat cardiac cell sheets without (left) and with the algae (right) on 60-mm polystyrene culture dishes (**B**).

Fourth, creatine kinase (CK) release from the cardiac tissues was measured to detect any damage of the cardiomyocytes. The release of CK from cultured cells is used as a common index of damage and death of muscle cells including cardiomyocytes^31^. CK release from a single-layer cardiac cell sheet without algae or with algae was 5.7 ± 0.2 U or 3.7 ± 3.2 U, respectively (n = 3). In the ten-layered cardiac cell sheets without algae, the significant release of CK, which was more than 40-folds that of the single cell sheet, was detected (Fig. 5A), indicating severe damage of cardiomyocytes within the tissues. However, the amount released from the layered cell sheets with algae was decreased to less than one-fifth (Fig. 5A). Additionally, 80% of the ten-layered cardiac cell sheets without algae were partially or completely detached from the culture dishes as shown in Fig. 5B within 6 days of cultivation (4 of 5 cases), suggesting the severe tissue damage. However, all ten-layered cell sheets with algae were continuing to attach to the dishes for the follow-up periods (6 days) (n = 5, Fig. 5B). These results showed that the severe cell damage within the multi-layered cell sheets was relieved by the co-cultivation with algae.

## Discussion

On the earth, “symbiotic relationships” exist between various green plants, algae, animals, and bacteria, and together they build a“recycling society”. This study showed the possibility of an *in vitro* symbiotic relationship between mammalian cells and algae. A recycling system was created in which algae supplied O_2_ to mammalian cells and in turn reused the metabolic waste products (CO_2_, ammonia) from mammalian cells, while mammalian cells used the O_2_, and excreted CO_2_ and metabolites. The culture conditions within thicker multi-cell layered tissues were improved by this co-culture system. In the cultivation of thicker cell-dense tissues without algae, cell damage occurred within the tissues (Fig. 4), which in turn induced anaerobic respiration (Table 2). In anaerobic respiration, only two molar ATP per one molar glucose are produced and lactate is also produced^1^. Inefficient anaerobic respiration induced active glucose consumption and the active production of lactate (Fig. 3). The toxic effect of lactate on cells (pH and osmolarity) occurs at a concentration greater than 20–30 mM, and cell growth is reduced by ammonia concentrations greater than 2–3 mM^25^. In this study, the culture media of five-layered and ten-layered cardiac cell sheets contained approximately 5 mM lactate and 0.16 mM ammonia (data not shown). On the other hand, the diffusional inhibition of their molecules may induce high concentrated accumulation of lactate and ammonia just after the production from dense mammalian cells, and severe glucose deficiency and hypoxia at the microenvironment of the cells. This adverse environment induced severe cytotoxicity and tissue damage, especially at the bottom part, which made direct contact with the dish^23^ (Fig. 4). In the case of the co-cultivation of mammalian cells and algae, the algae produced O_2_ even in the conditions (Fig. 2B,C). The oxygen supply induced aerobic respiration even within the cell-dense tissue (Table 2). In aerobic respiration, approximately 30 molar ATP are produced by the consumption of one molar glucose and no lactate is produced^1^. The effective respiration induces mild glucose consumption and inhibits active lactate accumulation. Additionally, in the co-culture system, ammonia might be rapidly consumed by algae as a source of amino acid-synthesis just after their production. The co-cultivation showed a significant decrease in glucose consumption and the accumulation of lactate and ammonia (Fig. 3). Histological observation and the release of CK showed that the condition of the cells within the tissues was improved by the co-cultivation (Figs 4 and 5). The supply of O_2_ from algae and the consumption of ammonia by algae are thought to be closely related to the improvement of cell condition within thicker tissues. Thicker cardiac tissues approximately 160 μm could be created by the co-culture system, even though it has been reported that the thickness limitation of tissues without vascular networks is approximately 40–80 μm^22^,^23^. A native-like bioengineered tissue is useful as an *in vitro* surrogate tissue model for estimating the efficacy and cytotoxicity of candidate drugs. At present, we are preparing to estimate electrophysiologically and functionally whether a heterogeneous cardiac tissue with algae can be used as a tissue model for pharmacology and toxicology. On the other hand, the supply of nutrients/biological components (hydrocarbon, amino acids, and lipids) from algae to mammalian cells is as yet unproven. If the supply of components from algae to mammalian cells can be induced by some kind of method, for example, genetic recombination technology, ultimately mammalian cells may be able to be cultured in very poor solutions like phosphate-buffered saline. Algae have the ability to store/secrete energy-rich hydrocarbons^32^. At present, genetically engineered algae are being researched to efficiently obtain useful materials, for example, petroleum substitutes, bioethanol, and biodiesel^32^^,^^33^. Those techniques offer a possible opportunity for the efficient production and secretion of hydrocarbons, lipids, and amino acids. Advances in this area of research may induce to the fabrication of millimeter-level thicker organ-like tissues and low-cost cell culture, which would have a great impact on cell biology, medicine, and industry.

In the culture medium without cells, ammonia could be detected (Fig. 3C) because it contained FBS and all mammalian blood, including bovine, contains ammonia. Additionally, it is thought that the degradation of amino acids, especially L-glutamine, in the medium causes the detection of ammonia. The half-life of L-glutamine in media with serum (37 °C) is 3–25 days^25^. After 1 day-cultivation, approximately 0.13 mM ammonia in M199-based medium containing 6% FBS for cardiac cells and 0.39 mM in Dulbecco’s modified Eagle medium (DMEM) containing 10% FBS for C2C12 cells were detected (data not shown). The ammonia concentration may negatively affect growth, biochemical reactions and gene expression of cultured cells. The amount of ammonia in the culture medium was significantly reduced by the co-cultivation with algae (Fig. 3C). The development of a practical co-culture system with algae will have a positive impact on the normal culture of mammalian cells.

In this study, a co-cultivation system with algae was applied to create thicker bioengineered cardiac tissues. Cell sheet technology has applied to create and regenerate various tissues, and clinical studies have already been performed successfully in six different fields, including cardiovascular medicine, gastrointestinal medicine, ophthalmology, periodontal disease, orthopedic surgery, and otolaryngology^10^,^12^,^13^,^14^,^15^,^16^,^17^,^18^. Additional clinical research in the field of respiratory surgery with cell sheet technology is now being prepared^34^. It is thought that 10^9^-level’s cells are necessary to treat just one patient with myocardial infarction or diabetes mellitus^35^. The creation of thicker healthy tissues allows us to transplant ever greater numbers of cells. The co-cultivation with algae allowed us to create multi-layered cell sheet-tissues (approximately 160 μm) at once under lights (Fig. 4). At present, we are preparing the transplantation of these multi-layered cell sheet-tissues into animal models. In some methods after transplantation, photoirradiation is necessary to maintain the thicker tissues *in vivo* until functional anastomoses with the host vasculature are formed. In a rat animal model, the functional anastomoses were rapidly formed within 12 hours after transplantation of cardiac cell sheet-tissues^36^. Because it was shown that the algae used in this study were damaged at 37 °C, the algae may also be damaged at the body temperature of a host. Our preliminary experiment showed that the algae did produce oxygen for several hours at 37 °C as like at 30 °C. On the other hand, some kinds of algae can be successfully cultured at 37 °C or even higher^37^. At present, we have started to co-culture thermostable algae and mammalian cells at 37 °C, and this system will contribute to the establishment of an optimal *in vitro* tissue-model as well as produce transplantable tissue. Inversely, the contamination of algae may have a problem after the formation of functional anastomoses with host vasculature. The thermosensitive algae used in this study could be removed gradually by the host body temperature so that only the target cells remain within the transplanted tissue. Moreover, at the time of tissue transplantation anti-algae antibody or the local application of non-toxic herbicides may be useful to be removed the algae from the tissue. Nevertheless, the contamination may be the cause of pathogenicity or immunogenicity after the transplantation. Recently, Schenck *et al*. reported about the transplantation of a bioartificial scaffold containing microalgae, *Chlamydomonas reinhardtii*, into a nude mouse full skin defect model or a zebrafish model^38^. In the experiment, no significant inflammatory response to the algae was observed in both mouse and zebrafish models. There are various kinds of algae. *in vivo* animal experiments, some algae will be selected as pathogenicity/immunogenicity-free algae. Concerning the *in vivo* transplantation of the heterogeneous tissue, more detailed investigation will be necessary.

This study proposed the possibility of an *in vitro* “symbiotic relationship” between mammalian cells and algae, and a “recycling system”, in which algae supply oxygen to mammalian cells, and reuse metabolites and waste products from mammalian cells; while mammalian cells consume the oxygen, and excrete metabolites and waste products. We believe that this new system has great potential for applications in the fields of cell biology, tissue engineering, and regenerative medicine.

## Methods

All animal experiments were performed according to the Guidelines of Tokyo Women’s Medical University on Animal Use, and consistent with the Guide for the Care and Use of Laboratory Animals prepared by the Institute of Laboratory Animal Resources (ILAR). All the experiments were approved by the Institutional Animal Care and Use Committee of Tokyo Women’s Medical University.

### Cell culture and cell sheet preparation

C2C12 mouse myoblast lines (Sumitomo Dainippon Pharma, Osaka, Japan) were cultured in DMEM (Sigma-Aldrich, St. Louis, MO, USA) supplemented with 10% FBS (Japan Bio Serum, Nagoya, Japan) and 1% penicillin-streptomycin (Invitrogen, Carlsbad, CA, USA), and C2C12 cell sheets were prepared according to previous reports^5^,^6^,^24^. Neonatal rat cardiac cells were isolated from the ventricles of 1-day-old Sprague-Dawley (SD) rats (CLEA, Tokyo, Japan), and cultured in an M199-based culture medium [40% Medium 199 (Invitrogen Corp., Carlsbad, CA, USA), 6% FBS, 0.2% penicillin-streptomycin, 2.7 mM glucose, and 54% balanced salt solution containing (in mM) 116 NaCl, 1.0 NaH_2_PO_4_, 0.8 MgSO_4_, 1.18 KCl, 0.87 CaCl_2_, and 26.2 NaHCO_3_], and a cardiac cell sheet was prepared as described in previous reports^5^,^6^,^39^,^40^,^41^.

### Algae culture

Eukaryotic algae, *Chlorococcum littorale*, were obtained from the Biological Resource Center, National Institute of Technology and Evaluation (Tokyo, Japan), and were cultured in Daigo’s IMK Medium (Wako Pure Chemical, Tokyo, Japan) with synthetic seawater (Wako Pure Chemical) at room temperature (approximately 25–28 °C) under continuous light (approximately 500–700 lux). The algae (2.5 × 10^7^ cells) were cultured in an M199-based culture medium at 30 °C or 37 °C under continuous light [1313 ± 45 lux (n = 3)] for 0 or 1 day on a 35-mm polystyrene culture dish (Corning, NY, USA), and then oxygen production was measured by an oxygen measurement system as described below. Illuminance was measured by illuminometer (As one, Osaka, Japan). Macroscopic photographs were taken by a digital camera (GR DIGITAL III, Ricoh, Tokyo, Japan).

### Co-cultivation of cell sheets and algae, and histological analysis

The co-cultivation of mammalian cell sheets and the algae was performed as shown in Table 1. Single- or multi-layered cell sheet-tissues were cultured at 30 °C in a humidified atmosphere with 5% CO_2_ under continuous light [1313 ± 45 lux (n = 3)] in the culture box (Supplementary Fig. 1B). Culture media were changed every day, and harvested culture media were used for analyzing the metabolic activity and cell viability of the tissues. Layered cell sheets were fixed with 4% paraformaldehyde solution (Muto Pure Chemicals, Tokyo, Japan). Specimens were embedded in paraffin, sectioned, and subjected to a histological examination by hematoxylin-eosin staining. Prepared specimens were observed by an optical microscope (ECLIPSE E800) (Nikon, Tokyo, Japan). The images were processed using an imaging system (NIS-Elements) (Nikon).

### Biochemical assay

The metabolic activities of cell sheet-tissues were monitored by measuring glucose, lactate, and ammonia concentrations in culture media. The cytotoxicity of cardiomyocytes was detected by CK release in culture media. The concentrations of glucose and lactate and CK activities were determined as described previously^31^,^42^,^43^,^44^. Total glucose consumption was calculated by subtracting the glucose concentration in the medium with cell sheets/algae or the cell sheets after the cultivation from the medium without cell sheets/algae after the cultivation. The values of lactate production and CK release were calculated by subtracting the backgrounds of their concentrations in the medium without cell sheets/algae or the cell sheets after the cultivation from the medium after the cultivation. Ammonia concentration was measured by a colorimetric method at an outsourcing laboratory, SRL (Tokyo, Japan).

### Measurement of oxygen concentrations in culture medium

Recently, we have developed a measurement system for oxygen concentration using an oxygen microelectrode sensor [a Clark-type oxygen microsensor with a 8–12 μm-diameter tip made of fragile glass (OX-10) (Unisense, Denmark)] and a high-precision electronic balance (HTR-220) (Shinko Denshi, Tokyo, Japan), which can detect the position of the tip of the oxygen sensor when it comes into contact with the bottom of the dish^27^,^28^,^29^. The measurement was performed in a humidified atmosphere containing 20% oxygen and 5% CO_2_ in a glove box hypoxia workstation (INVIVO2 300) (Ruskinn Technology, Mid Glamorgan, UK). Oxygen concentrations in culture dishes were estimated by the system with or without light [1103 ± 25 lux (n = 2)]. The measurements in Figs 1C and 2A,B were performed at three points, and the two independent experiments were also performed. The representable profiles were shown in the figures.

### Data analysis

Data in Figs 2C, 3 and 5A are expressed as means ± standard deviation. An unpaired Student *t*-test was performed to compare two groups. One-way analysis of variance (Ryan’s method) was used for multiple group comparisons. A *p*-value of less than 0.05 was considered significant.

## Additional Information

**How to cite this article**: Haraguchi, Y. *et al*. Thicker three-dimensional tissue from a “symbiotic recycling system” combining mammalian cells and algae. *Sci. Rep.* **7**, 41594; doi: 10.1038/srep41594 (2017).

**Publisher's note:** Springer Nature remains neutral with regard to jurisdictional claims in published maps and institutional affiliations.

## Supplementary Material

Supplementary Figure 1

## Figures and Tables

**Table 1:** Cells and media used in this study.

Figures	Cells	Cell numbers	Media	Medium volume	Culture temperature
Fig. 1C	*Chlorococcum littorale*	2.5 × 10^7^ cells	M199-based medium	2 mL	30 °C
Fig. 2A	C2C12 cell sheet	Single-layer cell sheet	Dulbecco’s modified Eagle medium	6 mL	30 °C
Fig. 2B,C	C2C12 cell sheet	Single-layer cell sheet	Dulbecco’s modified Eagle medium	6 mL	30 °C
	*Chlorococcum littorale*	2.5 × 10^7^ cells			
Figs 2C, and 3A,B	Rat cardiac cell sheet	Single-layer cell sheet	M199-based medium	6 mL	30 °C
	*Chlorococcum littorale*	2.5 × 10^7^ cells			
Figs 3, 4, 5	Rat cardiac cell sheet	Five-or ten-layered cell sheets	M199-based medium	6 mL	30 °C
	*Chlorococcum littorale*	2.5 × 10^8^ cells			

**Table 2:** The ratios of lactate production to glucose consumption in the medium after cultivation for 24 h.

Cardiac cell sheets	Lactate-to-glucose ratio (mol/mol)
Single-layer without algae	1.40 ± 0.07^a,b^ (n = 3)
Single-layer with algae	1.36 ± 0.07 (n = 3)
Five-layer without algae	1.82 ± 0.04^a,c^ (n = 3)
Five-layer with algae	1.41 ± 0.20^c^ (n = 3)
Ten-layer without algae	1.71 ± 0.14^b,d^ (n = 5)
Ten-layer with algae	1.40 ± 0.14^d^ (n = 5)

^a,b,c,d^*p* < 0.05.

## References

[b1] Albert B. . Energy conversion: mitochondria and chloroplasts. In Molecular biology of the cell, Vol. 6 (eds Albert B. . ) Ch. 14, 753–812 (Garland Science, 2015).

[b2] Haraguchi Y., Shimizu T., Yamato M. & Okano T. Cell therapy and tissue engineering for cardiovascular disease. Stem Cells Transl. Med. 1, 136–141 (2012). 23197760 10.5966/sctm.2012-0030 PMC3659688

[b3] Behfar A., Crespo-Diaz R., Terzic A. & Gersh B. J. Cell therapy for cardiac repair-lessons from clinical trials. Nat. Rev. Cardiol. 11, 232–246 (2014). 24594893 10.1038/nrcardio.2014.9

[b4] Okano T., Yamada N., Sakai H. & Sakurai Y. A novel recovery system for cultured cells using plasma-treated polystyrene dishes grafted with poly (*N*-isopropylacrylamide). J. Biomed. Mater. Res. 27, 1243–1251 (1993). 8245039 10.1002/jbm.820271005

[b5] Haraguchi Y. . Fabrication of functional three-dimensional tissues by stacking cell sheets *in vitro*. Nat. Protoc. 7, 850–858 (2012). 22481530 10.1038/nprot.2012.027

[b6] Haraguchi Y. . Cell sheet technology for cardiac tissue engineering. Methods Mol. Biol. 1181, 139–155 (2014). 25070334 10.1007/978-1-4939-1047-2_13

[b7] Memon I. A. . Repair of impaired myocardium by means of implantation of engineered autologous myoblast sheets. J. Thorac. Cardiovasc. Surg. 130, 1333–1341 (2005). 16256786 10.1016/j.jtcvs.2005.07.023

[b8] Sekine H. . Cardiac cell sheet transplantation improves damaged heart function via superior cell survival in comparison with dissociated cell injection. Tissue Eng. Part A 17, 2973–2980 (2011). 21875331 10.1089/ten.tea.2010.0659

[b9] Chang D. . Time course of cell sheet adhesion to porcine heart tissue after transplantation. PLoS One 10 e0137494 (2015). 26444683 10.1371/journal.pone.0137494 PMC4596823

[b10] Nishida K. . Corneal reconstruction with tissue-engineered cell sheets composed of autologous oral mucosal epithelium. N. Engl. J. Med. 351, 1187–1196 (2004). 15371576 10.1056/NEJMoa040455

[b11] Haraguchi Y., Shimizu T., Yamato M. & Okano T. Regenerative therapies using cell sheet-based tissue engineering for cardiac disease. Cardiol. Res. Pract. 2011, 845170 (2011). 22007333 10.4061/2011/845170 PMC3189561

[b12] Sawa Y. . Tissue engineered myoblast sheets improved cardiac function sufficiently to discontinue LVAS in a patient with DCM: report of a case. Surg. Today 42, 181–184 (2012). 22200756 10.1007/s00595-011-0106-4

[b13] Burillon C. . Cultured autologous oral mucosal epithelial cell sheet (CAOMECS) transplantation for the treatment of corneal limbal epithelial stem cell deficiency. Invest. Ophthalmol. Vis. Sci. 53, 1325–1331 (2012). 22064987 10.1167/iovs.11-7744

[b14] Ohki T. . Prevention of esophageal stricture after endoscopic submucosal dissection using tissue-engineered cell sheets. Gastroenterology 143, 582–588 (2012). 22561054 10.1053/j.gastro.2012.04.050

[b15] Sato M., Yamato M., Hamahashi K., Okano T. & Mochida J. Articular cartilage regeneration using cell sheet technology. Anat. Rec. 297, 36–43 (2014). 10.1002/ar.22829 24293096

[b16] Egami M., Haraguchi Y., Shimizu T., Yamato M. & Okano T. Latest status of the clinical and industrial applications of cell sheet engineering and regenerative medicine. Arch. Pharm. Res. 37, 96–106 (2014). 24293063 10.1007/s12272-013-0299-8

[b17] Iwata T. . Cell sheet engineering and its application for periodontal regeneration. J. Tissue Eng. Regen. Med. 9, 343–356 (2015). 23881816 10.1002/term.1785

[b18] Yaguchi Y. . Middle ear mucosal regeneration with three-dimensionally tissue-engineered autologous middle ear cell sheets in rabbit model. J. Tissue Eng. Regen. 10, E188–194 (2016). 10.1002/term.1790 23894137

[b19] Abbott A. Biology’s new dimension. Nature 424, 870–872 (2004). 10.1038/424870a 12931155

[b20] Kaneshiro N. . Bioengineered chondrocyte sheets may be potentially useful for the treatment of partial thickness defects of articular cartilage. Biochem. Biophys. Res. Commun. 349, 723–731 (2006). 16949051 10.1016/j.bbrc.2006.08.096

[b21] Mitani G. . The properties of bioengineered chondrocyte sheets for cartilage regeneration. BMC Biotechnol. 9, 17 (2009). 19267909 10.1186/1472-6750-9-17 PMC2662823

[b22] Shimizu T. . Polysurgery of cell sheet grafts overcomes diffusion limits to produce thick, vascularized myocardial tissues. FASEB J. 20, 708–710 (2006). 16439619 10.1096/fj.05-4715fje

[b23] Sekine W., Haraguchi Y., Shimizu T., Umezawa A. & Okano T. Thickness limitation and cell viability of multi-layered cell sheets and overcoming the diffusion limit by a porous-membrane culture insert. J. Biochip. Tissue chip. S2, 001 (2011).

[b24] Haraguchi Y. . Development of a new assay system for evaluating the permeability of various substances through three-dimensional tissue. Tissue Eng. Part C Methods 16, 685–692 (2010). 19788345 10.1089/ten.TEC.2009.0459

[b25] Schneider M., Marison I. W. & von Stockar U. The importance of ammonia in mammalian cell culture. J. Biotechnol. 46, 161–185 (1996). 8672289 10.1016/0168-1656(95)00196-4

[b26] Pesheva I., Kodama M., Dionisio-Sese M. L. & Miyachi S. Changes in photosynthetic characteristics induced by transferring air-grown cells of *Chlorococcum littorale* to high-CO_2_ conditions. Plant Cell Physiol. 35, 379–387 (1994).

[b27] Sekine K. . Oxygen consumption of human heart cells in monolayer culture. Biochem. Biophys. Res. Commun. 452, 834–839 (2014). 25218502 10.1016/j.bbrc.2014.09.018

[b28] Kagawa Y., Matsuura K., Shimizu T. & Tsuneda S. Direct measurement of local dissolved oxygen concentration spatial profiles in a cell culture environment. Biotechnol. Bioeng. 112, 1263–1274 (2015). 25565074 10.1002/bit.25531

[b29] Kagawa Y., Haraguchi Y., Tsuneda S. & Shimizu T. Real-time quantitation of internal metabolic activity of three-dimensional engineered tissues using an oxygen microelectrode and optical coherence tomography. J. Biomed. Mater. Res. B Appl. Biomater. doi: 10.1002/jbm.b.33582. 26821598

[b30] Nakano S. . A usage of CO_2_ hydrate: Convenient method to increase CO_2_ concentration in culturing algae. Bioresour. Technol. 172, 444–448 (2014). 25263943 10.1016/j.biortech.2014.09.019

[b31] Shioyama T., Haraguchi Y., Muragaki Y., Shimizu T. & Okano T. New isolation system for collecting living cells from tissue. J. Biosci. Bioeng. 115, 100–103 (2013). 23063341 10.1016/j.jbiosc.2012.08.013

[b32] Radakovits R., Jinkerson R. E., Darzins A. & Posewitz M. C. Genetic engineering of algae for enhanced biofuel production. Eukaryot. Cell 9, 486–501 (2010). 20139239 10.1128/EC.00364-09 PMC2863401

[b33] Oliver J. W., Machado I. M., Yoneda H. & Atsumi S. Cyanobacterial conversion of carbon dioxide to 2,3-butanediol. Proc. Natl. Acad. Sci. USA 110, 1249–1254 (2013). 23297225 10.1073/pnas.1213024110 PMC3557092

[b34] Kanzaki M. . Functional closure of visceral pleural defects by autologous tissue engineered cell sheets. Eur. J. Cardiothorac. Surg. 34, 864–869 (2008). 18586511 10.1016/j.ejcts.2008.05.048

[b35] Zweigerdt R., Olmer R., Singh H., Haverich A. & Martin U. Scalable expansion of human pluripotent stem cells in suspension culture. Nat. Protoc. 6, 689–700 (2011). 21527925 10.1038/nprot.2011.318

[b36] Takeuchi R. . *In vivo* vascularization of cell sheets provided better long-term tissue survival than injection of cell suspension. J. Tissue Eng. Regen. Med. 10, 700–710 (2016). 24470393 10.1002/term.1854

[b37] Sano S. . Characterization of ascorbate peroxidases from unicellular red alga *Galdieria partita*. Plant Cell Physiol. 42, 433–440 (2001). 11333315 10.1093/pcp/pce054

[b38] Schenck T. L. . Photosynthetic biomaterials: A pathway towards autotrophic tissue engineering. Acta Biomater. 15, 39–47 (2015). 25536030 10.1016/j.actbio.2014.12.012

[b39] Haraguchi Y., Shimizu T., Yamato M., Kikuchi A. & Okano T. Electrical coupling of cardiomyocyte sheets occurs rapidly via functional gap junction formation. Biomaterials 27, 4765–4774 (2006). 16737736 10.1016/j.biomaterials.2006.04.034

[b40] Sekine H. . *In vitro* fabrication of functional three-dimensional tissues with perfusable blood vessels. Nat. Commun. 4, 1399 (2013). 23360990 10.1038/ncomms2406 PMC3660653

[b41] Sakaguchi K. . *In vitro* engineering of vascularized tissue surrogates. Sci. Rep. 3, 1316 (2013). 23419835 10.1038/srep01316 PMC3575583

[b42] Tadakuma K. . Development of a simple device for transfer/transplantation of living cell sheets rapidly and completely without cell damage. Biomaterials 34, 9018–9025 (2013). 23972478 10.1016/j.biomaterials.2013.08.006

[b43] Haraguchi Y., Matsuura K., Shimizu T., Yamato M. & Okano T. Simple suspension culture system of human iPS cells maintaining their pluripotency for cardiac cell sheet engineering. J. Tissue Eng. Regen. Med. 9, 1363–1375 (2015). 23728860 10.1002/term.1761

[b44] Hasegawa A., Haraguchi Y., Shimizu T. & Okano T. Rapid fabrication system of three-dimensional tissues by cell sheet technology and centrifugation. J. Biomed. Mater. Res. A 103, 3825–3833 (2015). 26097136 10.1002/jbm.a.35526

