# Supplementary material for: Thicker three-dimensional tissue from a “symbiotic recycling system” combining mammalian cells and algae
Source: Sci Rep. 2017 Jan 31;7:41594. doi: 10.1038/srep41594 (PMC5282507; doi:10.1038/srep41594)

Thicker three-dimensional tissue from a "symbiotic recycling system" combining mammalian cells and algae

Yuji Haraguchi, Yuki Kagawa, Katsuhisa Sakaguchi, Katsuhisa Matsuura,

Tatsuya Shimizu, Teruo Okano

Supplementary figure legend

Supplementary Figure 1 | Co-culture system of mammalian cells and algae, *Chlorococcum littorale*. The algae were inserted in the multi-layered cell sheets as shown schematically in (A). C2C12 cell sheets or rat cardiac cell sheets with/without algae were cultured at 30 °C under continuous light (B).

Supplementary Figure 1

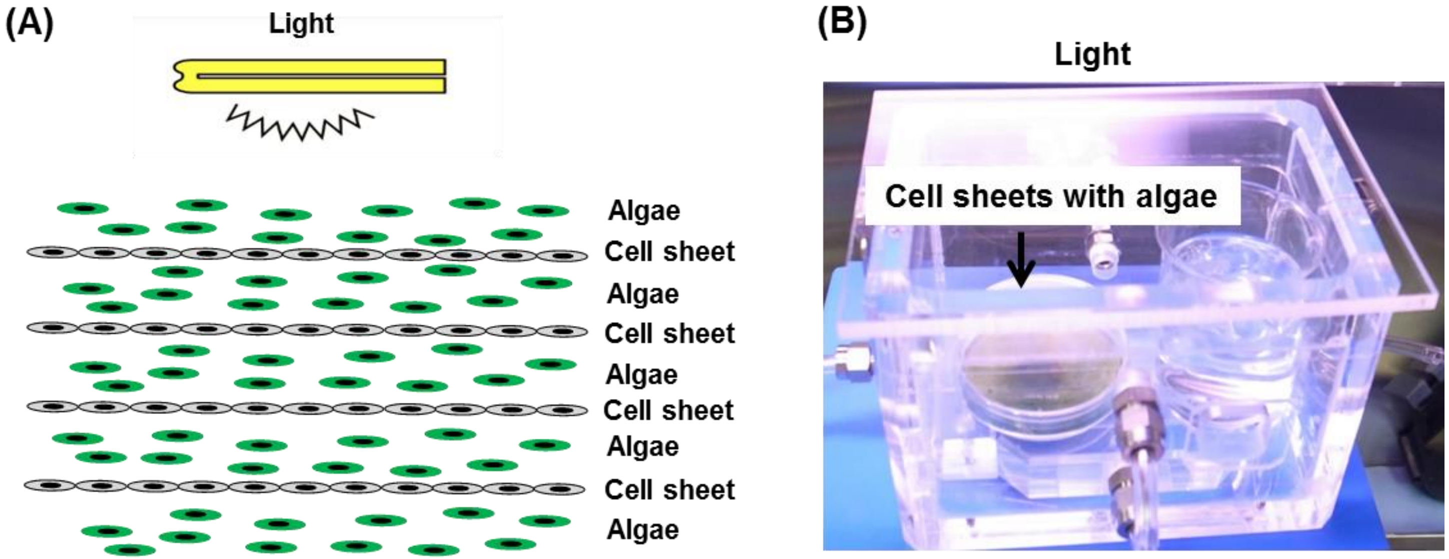

Supplement: Supplementary Figure 1 [file srep41594-s1.pdf]
